# Impacts of Post-Covid Condition (PCC) in Sweden: a cross-sectional observational survey study

**DOI:** 10.1186/s12889-026-27720-7

**Published:** 2026-05-12

**Authors:** Filip Jovicic, Lance M. McCracken, Alexander Rozental, Monica Buhrman

**Affiliations:** 1 Department of Psychology, Uppsala University, P.O. Box 1225, von Kraemers Allé 1A, SE- 752 37 Uppsala, Sweden; 2 Department of Health, Education and Technology, Luleå University of Technology, Laboratorievägen 14, Luleå, SE-971 87 Sweden; 3 Department of Clinical Neuroscience, Karolinska Institutet, Retzius väg 8, Solna, SE-171 65 Sweden

**Keywords:** Post-Covid Condition, COVID-19, Long covid, Pandemic, Health impact, Daily functioning, Satisfaction with life, Behavioral medicine

## Abstract

**Background:**

The COVID-19 pandemic left many people with persistent health problems following a COVID-19 infection. Research on the condition better known as Post-Covid Condition (PCC) is still inconclusive, and few evidence-based treatments are currently available, although several studies report promising results. There are however people in need of treatment and support.

**Methods:**

To better understand what such help might entail, we conducted an online-based survey (*n* = 629, 82.6% women) to examine the impact of PCC in Sweden and explore relationships among various variables using correlation analysis. Swedish-speaking adults with at least one persisting health problem after COVID-19 were included in the study. The survey included demographic and psychosocial variables, historic COVID-19 data, other clinical measures of psychological distress, satisfaction with life, and daily functioning impairment.

**Results:**

Results indicate a substantial and heterogeneous impact of PCC in this sample. Participants reported an average of 10.70 (SD = 5.62) symptoms with the most frequently reported being fatigue (90.9%, *n* = 507) and cognitive deficits (73.3%, *n* = 409). The highest average symptom burden scores were reported for post-exertional malaise (PEM; M = 8.37, SD = 1.70) and fatigue (M = 8.22, SD = 1.77). Medication, self-help advice, and physiotherapy were the most frequently reported treatment modalities. Most participants reported their problems being unchanged or improved after treatment, but there were reports of worsening as well. Regarding psychological distress, participants reported mild anxiety, moderate depression, and subclinical but notable levels of sleeping problems. On average, daily functioning was substantially impaired by PCC and participants reported being “somewhat dissatisfied with life”. The correlational analyses revealed several significant correlations (|*r*| = 0.11-0.93, *p* < .01), with strongest relationships between the clinical variables.

**Conclusions:**

While this study aimed to measure impacts of PCC in many different areas of life, it holds some limitations, and there are many questions remaining. We present several recommendations for methodology and future research topics. Identifying modifiable variables that can be used in developing a treatment is naturally a next step. Based on empiric evidence from similar conditions, psychological flexibility (PF) may be a promising one.

**Supplementary Information:**

The online version contains supplementary material available at 10.1186/s12889-026-27720-7.

## Background

More than five years have passed since COVID-19 was classed as a pandemic and a threat to people’s health by the World Health Organization (WHO). COVID-19 had a significant impact on society, and several years after the outbreak impacts remain, including persistent health problems following COVID-19 infections for some people. This is better known as post-covid condition (PCC), although several different terms are also used [1]. PCC is defined by WHO as having at least one, up to as many as 25, new-onset- or persisting symptoms, three months after a COVID-19 infection, and lasting for at least two months. Common symptoms include fatigue, cognitive impairments, and shortness of breath, but can also include allergies, dizziness, and impaired hearing [2]. As a result of multiple definitions, differences in PCC symptoms, and severity thresholds, prevalence estimates vary. Based on a subset of six studies in a systematic review and meta-analysis, estimated prevalence for having at least one PCC symptom is over 50% up to two years following the infection for those who experience symptoms three months after a COVID-19 infection [3]. The certainty of this evidence, however, is low. There are many remaining questions concerning true prevalence, cause, underlying pathology, individual susceptibility, factors underlying persistence of PCC, as well as how it can be treated.

PCC is characterized by a heterogeneous set of symptoms that appear multisystemic, meaning multiple organ systems are included [4, 5]. Due to the complex clinical manifestations, and the potential for multiple pathophysiological mechanisms, there is ongoing debate on whether PCC is one condition or several [1]. Putative biomedical models of PCC include dysregulations in the immune system, or effects of persistent viral proteins, leading to damage in tissues and organs, although the clinical significance of these observed abnormalities is unclear and evidence is equivocal. It is commonly believed that neurological symptoms of PCC are necessarily accompanied by damage in the nervous system, but this too is questioned [6]. A recent study suggests PCC can be divided into four subtypes [7], although this is not yet supported by evidence.

As for the importance of psychological variables in PCC, a recently published systematic review found that, while current evidence of psychological factors in PCC is inconclusive, depression and anxiety seem to be highly associated with the condition and may be relevant targets of multidisciplinary treatments for the condition [8]. More research is needed to determine what role psychosocial variables or a person’s behavior might play in PCC, if any. Naturally, the role could be in onset, persistence, severity, or impact. If there is a role, this could provide opportunities to intervene.

Although there are symptom-alleviating interventions for PCC with promising results (e.g. [9–11]), there are few treatments available to date with high certainty of evidence, including medical or psychological. The latter could be useful to support the wellbeing and daily functioning of those living with the condition, at the very least, given that such benefits are demonstrated across an exceptionally wide range of physical health conditions [12]. In a “living” systematic review investigating the effectiveness of interventions for adults with PCC, cognitive behavioral therapy (CBT), and combined physical and mental rehabilitation appear possibly effective, with moderate certainty. This includes suggested benefits for fatigue, concentration, depression, overall health, and quality of life [13]. Moderate aerobic training may be beneficial for this population [14] and exercise of varying intensity appears generally tolerated by people with PCC [15]. This suggests that behavior change and engagement in daily activity, including physical activity, may be a safe and a therapeutic avenue, at least for some [16].

In summary, research into PCC is steadily growing but inconclusive. Particularly, a role for psychological variables seems understudied. While prevalence numbers vary substantially depending on definitions of the condition, it seems highly likely that there is a considerable number of people in need of treatment. To move forward there is a need to understand prevalence of PCC and its impacts in the lives of people affected, including their daily functioning and wellbeing, and factors that influence these. Provided there is demonstrated need, and amenable targets for treatment, further work to develop treatments could then proceed.

The purpose of the current study is to assess and document the reporting of PCC and its impact, as well as the relationship between PCC symptom severity and psychosocial variables. Specific objectives are to describe: (a) demographic characteristics of the PCC population, (b) symptom frequencies, and impact on people’s health and functioning from PCC, and (c) factors associated with symptoms, symptom burden, psychological distress, satisfaction with life (SWL), and functional impairment.

## Methods

### Study design

This study is part of the *TACTIC* project [17] aiming to eventually develop a treatment focusing on increasing daily functioning and wellbeing for people with PCC. The current study aims to examine the experience of adults self-identified as having PCC and to document the symptoms and impact of this condition with a cross-sectional, observational, survey design.

### Participants and procedure

**Fig. 1:**
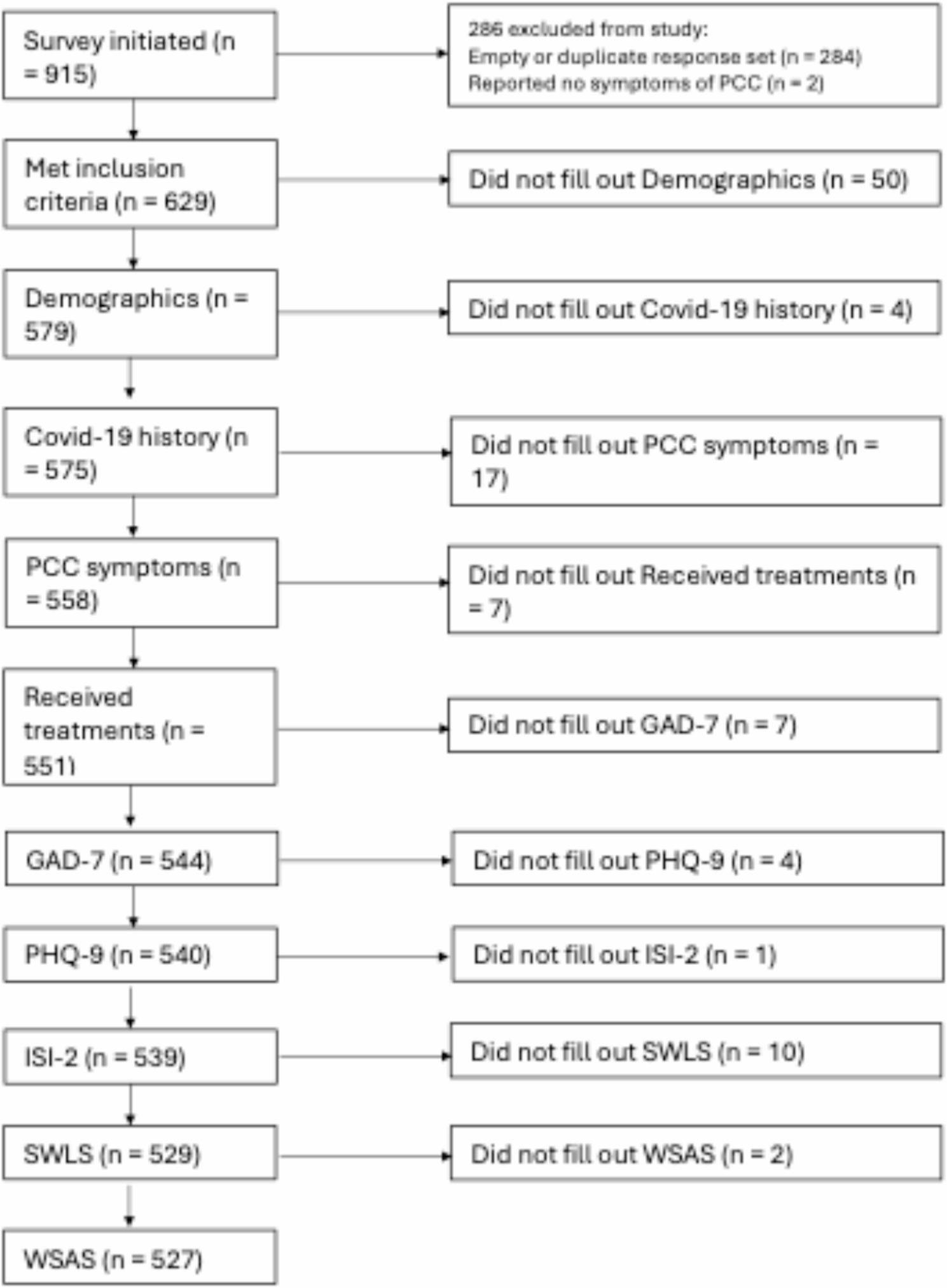
Flow chart over participant enrollment in study and analyses

Participants were eligible for the study if they (a) were ≥ 18 years old, (b) could read and write in Swedish, (c) had access to the technical equipment needed to fill out the survey, and (d) were bothered by at least one new or persisting health problem (symptom) after having been infected with the COVID-19 virus. Participants were excluded if they (a) did not provide informed consent, (b) were identified as a duplicate respondent, or (c) reported no PCC symptoms. In cases where duplicates were identified, the least complete response set was excluded. In duplicate cases where progress was equal, the most recent set of responses was included. A recruitment flowchart is provided in Fig. 1.

Recruitment took place between 26 October 2024 and 31 March 2025, using social media (Facebook including patient groups, Instagram, X, Reddit) and traditional notice-board advertising on Uppsala University campus. In addition, 103 health care clinics from 18 of the 21 Swedish regions helped with recruitment by posting advertisements in their waiting rooms. We know that 915 initial contacts were made with the survey, and the final sample consisted of 629 participants from 64% (*N* = 186) of the municipalities in Sweden. Informed consent was obtained from all the participants. Participants were informed about potential risks, that participation was voluntary and that they could withdraw at any time. No compensation was provided for participating in the study. The study was approved by the Swedish Ethical Review Authority (2024-04666-01), and was conducted in accordance with the ethical principles of research involving human participants in the Declaration of Helsinki. 

### Measures

A section-divided survey was created using Research Electronic Data Capture (RedCAP; [18, 19]) hosted at Uppsala University. The survey was developed for this study and consisted of several established measures as well as specific questions formulated for the purpose of this study, all divided into sections. An English translation of the survey items developed for this study is available in an additional document file [see Additional file 1]. The sections included questions about demographics, COVID-19, PCC symptoms and symptom burden, psychological distress, treatment received for COVID-19, SWL, functioning, psychological flexibility (PF), and open-ended qualitative items asking about participants views on causes and maintaining factors for their condition, and on what type of help they feel they need. Analyses of the PF and qualitative data are not included in this article and will be reported elsewhere.

All items in the survey were mandatory, meaning that participants could not proceed to the next section without completing the previous one. However, if the survey was exited prematurely, partial responses were retained. Consequently, missing data occurred, and sample sizes varied both across sections and within individual sections. However, comparisons revealed no meaningful differences between completers (63.8%, *n* = 401) and non-completers (36.2%, *n* = 228). Of the 18 variables included in the analysis, statistically significant differences were observed only for depression, *F*(1, 233) = 6.29, *p* < .05, and functional impairment, *F*(1, 193) = 8.12, *p* < .05. Post-hoc tests showed that mean differences were below clinically significant change thresholds for both depression (1.48 points on the PHQ-9), and functional impairment (3.13 on WSAS). While we cannot rule out with certainty that missing data were non-systematic, the difference between completers and non-completers does not seem to constitute a clinically meaningful difference, at least when it comes to levels of depression and functional impairment.

#### Demographics

The demographic section of the survey included items assessing gender, age, highest level of education, marital status, number of children, occupational status, city or town of residence, subjective financial status [20], as well as the extent and duration of sick leave. Participants entered their place of residence and responses were subsequently coded according to the Swedish Association of Local Authorities and Regions municipal classification system [21].

#### COVID-19 infection

COVID-19 questions included number of COVID-19 infection episodes, how the infection was verified, number of bedridden days, and if hospital treatment was received.

#### PCC symptoms

Participants were asked to mark every symptom they experienced during at least two months, and that arose or persisted at least three months after the COVID-19 infection. The 25 symptoms in the list, as well as the timeframe, were selected according to the WHO definition of PCC [2]. The list also allowed participants to choose a “no symptoms” or “other” option. For every symptom marked, participants were presented with an additional item, asking them to enter from 0 to 10 how much they were affected by the symptom where 0 indicated “not at all” and 10 “extremely”, resulting in a symptom burden variable for every PCC symptom. The total individual symptom burden was calculated by summing the symptom burden scores into a composite variable, ranging from 0 to 250.

#### Received treatment

In the section of the survey on treatment, participants were asked to estimate an approximate number of health care visits they had made related to PCC during the last six months. Then, they were asked to select from a list all types of treatment they have received for PCC. For every selected treatment, a follow-up question was presented asking participants how their problems had changed after the specific treatment ranging from “0 – very much improved” to “6 – very much worsened” with 3 reflecting “unchanged.” This follow-up question was based on items used to assess Patient Global Impression of Change (PGIC; [22]).

#### Psychological distress

Anxiety symptoms were measured with the Swedish version of the Generalized Anxiety Disorder-7 (GAD-7; [23]). It is a widely used, and considered reliable and valid measure of anxiety [24]. The GAD-7 consists of 7 items measuring anxiety, scored from 0 to 3. Total scores between 0 and 5 points correspond to no anxiety, 6 and 10 to mild anxiety levels, 11 and 15 moderate levels and scores between 16 and 21 points are interpreted as severe levels of anxiety. Significant anxiety is typically indicated by scores of 10 points or more. In this sample, internal consistency was *α* = 0.90.

Symptoms of depression were measured using the Swedish version of the Patient Health Questionnaire (PHQ-9), a measure of depressive symptoms based on the DSM-IV criteria for depression. It is considered reliable and valid in its original form [25]. It consists of 9 items, scored from 0 to 4. Total scores of 0–4 indicate no depression, 5–9 mild depression, 10–14 moderate depression, 15–19 moderately severe, and 20–27 severe depression. Significant depression is typically indicated by scores of 10 points or more. In this sample, internal consistency was *α* = 0.84.

Symptoms of insomnia were measured using a two-item version of the Swedish translation of the Insomnia Severity Index (ISI-2; [26]). The items ask participants about how satisfied they are with their sleep, and how much their current sleeping pattern interferes with their daily functioning, and are scored from 0 to 4. When using a 6 points cutoff, the 2-item version identifies insomnia disorder with comparable sensitivity/specificity scores as the full-length measure [26]. The full-length version of the ISI [27] is a reliable, valid and change sensitive measure for insomnia [28]. In this sample, internal consistency was *α* = 0.84.

#### Satisfaction with life

Satisfaction with life (SWL) was measured using a Swedish translation of the Satisfaction with Life Scale (SWLS; [29]). The measure consists of five items scored from 1 to 7 where 1 indicates “strongly disagree” and 7 indicates “strongly agree”. A total score of 5–9 indicates being “extremely dissatisfied”, 10–14 “dissatisfied”, 15–19 “slightly dissatisfied”, 20 “neutral”, 21–25 “slightly satisfied”, 26–30 “satisfied”, and 31–35 “very satisfied” [30]. The Swedish version of SWLS is considered reliable, though it appears unable to identify extreme levels of satisfaction [31]. In this sample, internal consistency was *α* = 0.89.

#### Functional impairment

Participants’ functioning was measured using the Swedish version of the Work and Social Adjustment Scale (WSAS; [32]) which is a measure of functional impairment in five domains of life: work/studies, activities in daily life, social activities, free time, and family and relationships. The five items are scored from 0 to 8, where 0 indicates “not at all reduced ability” and 8 indicates “extreme difficulties”. A total score of 20 or more has been suggested to correspond to severe functional impairment, although this cutoff originally is based on people with mental health or psychiatric problems [32]. However, WSAS scores have been shown to correlate negatively with physical functioning in a chronic fatigue sample and is considered to be a reliable and valid measure in this population [33]. In this sample, internal consistency was *α* = 0.91.

### Statistical analyses

Data were cleaned and recoded in Apple Numbers version 14.4. The statistical analyses were performed in jamovi version 2.3.28. Unanswered questions, such as in cases where participants closed the survey before it was completed, were treated as missing data and excluded from the analysis. Outliers in the variables “number of bedridden days” (*n* = 2) and “number of COVID-19 episodes” (*n* = 1) were identified and excluded based on thresholds reflecting values considered practically implausible or highly unlikely. The relationships between demographic variables, COVID-19 history and severity, PCC symptoms and symptom burden, psychological distress, SWL, and functioning were examined using Pearson’s product-moment correlation analyses. Several variables were dichotomized for these analyses as many of them were purely categorical, neither ordinal or continuous, and were readily suited to meaningful dichotomous recoding. This avoided analyses of categories with few participants and facilitated utilization of uniform statistical methods. An alpha level of < 0.01 was used to account for the relatively large sample size and to reduce the risk of inflating the findings.

## Results

### Demographics

The demographics section was completed by 579 participants. Demographics are presented in detail in Table 1. Participants were between 20 and 85 years old (M = 53.9, SD = 11.7). Most of the participants lived in medium- or large-sized city areas, were in a relationship, had some form of occupation, reported good or very good financial status and were highly educated.

**Table 1:** Participant demographics

Characteristics	*n*	%
Gender		
Woman	481	82.6
Man	96	16.5
Non-binary	3	0.5
Unsure	1	0.2
Prefer not to answer	1	0.2
Highest level of education		
Elementary	19	3.3
High school	129	22.2
Higher vocational education	57	9.8
Courses, university level	66	11.3
Bachelor	175	30.1
Master	118	20.3
PhD	12	2.1
Folk high school (non-formal adult education)	3	0.5
Other	3	0.5
Marital status		
Single	135	23.2
Partnered, living together	98	16.8
Partnered, not living together	36	6.2
Married	263	45.2
Divorced	41	7.0
Widow/widower	9	1.5
Occupational status		
Unemployed	35	6.0
Student	13	2.2
Employed	289	49.7
Self-employed	32	5.5
Parental leave	0	0.0
Disability pension	25	4.3
On sick leave for a longer period of time (> 60 days)	95	16.4
Job training or similar via e.g. Swedish Public Employment Service	2	0.3
Retired	90	15.5
Other	0	0.0
Sick leave, extent		
No	417	71.8
Yes, 25%	30	5.2
Yes, 50%	33	5.7
Yes, 75%	10	1.7
Yes, 100%	91	15.7
Sick leave, duration^a^		
Less than 2 weeks	2	1.2
2 weeks or longer	2	1.2
1 month or longer	0	0
2 months or longer	5	3.0
3 months or longer	6	3.7
6 months or longer	11	6.7
12 months or longer	138	84.1
Self-perceived financial status		
Very poor	25	4.3
Poor	64	11.0
Sufficient	182	31.3
Good	228	39.2
Very good	82	14.1
Place of residence		
Smaller towns/urban areas and rural municipalities	157	27.1
Medium-sized towns and municipalities near medium-sized towns	257	44.3
Large cities and municipalities near large cities	166	28.6

For variables gender, highest level of education and marital status, *n* = 582; sick leave extent, financial status and occupational status, *n* = 581; place of residence, *n* = 580

^a^ Only participants who were on sick leave (*n* = 164) to some extent received this question

### COVID-19 infection

Of the 575 participants who completed the COVID-19 infection section, 97.2% (*n* = 559) claimed they had COVID-19, 2.8% (*n* = 16) were unsure, and none said they never had COVID-19. Participants reported an average number of 2.27 (SD = 1.24) COVID-19 episodes. A clear majority, 87.5% (*n* = 503) reported they had been bedridden due to COVID-19 at some point, for a median number of 14 days (*n* = 573, range 0 to 1,825). Of the 90 participants who reported they received hospital care, 27.8% (*n* = 25) reported they were in the intensive care unit (ICU). 

### PCC symptoms

**Fig. 2:**
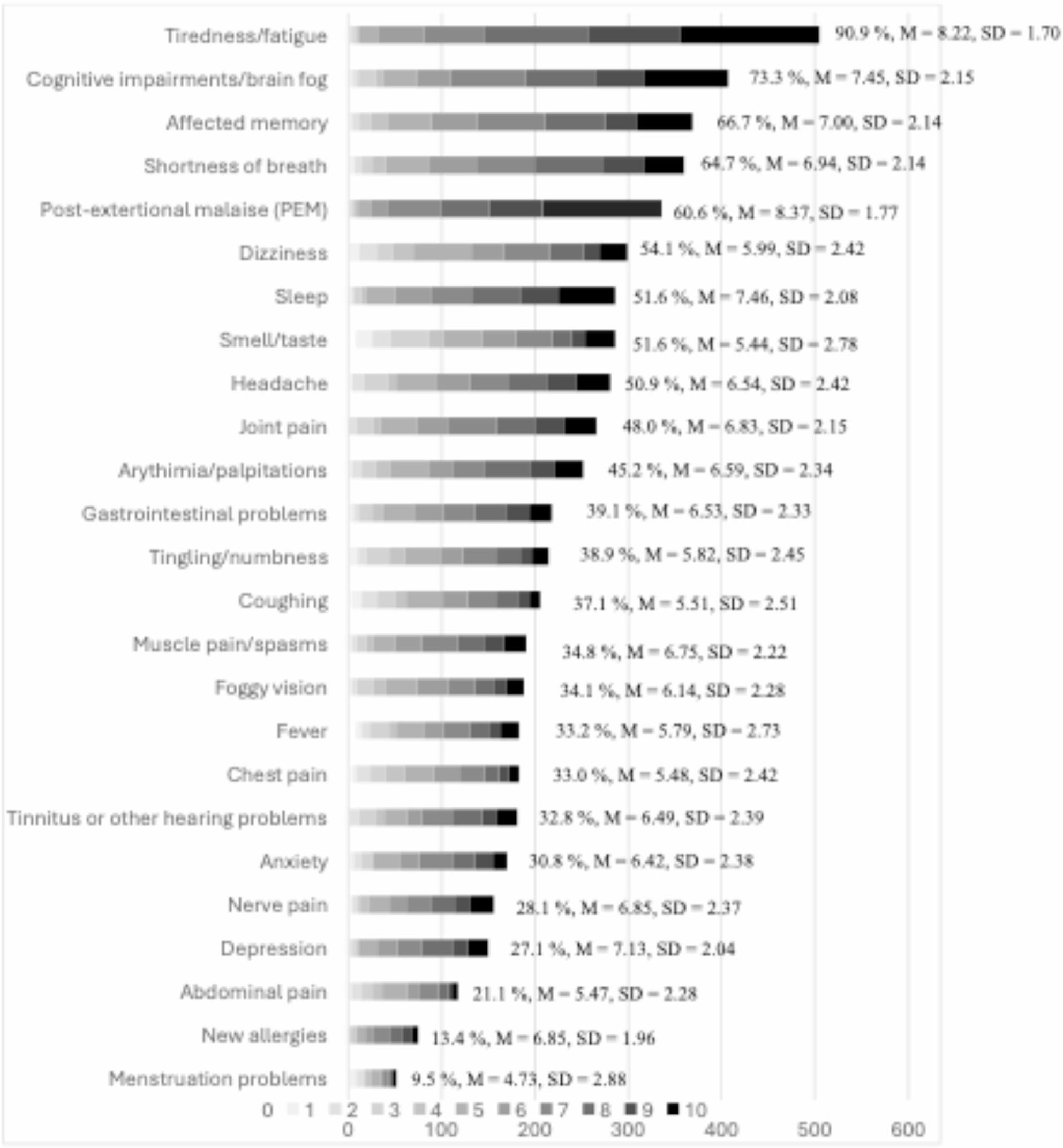
Frequencies of symptoms and distributions of means (M) and standard deviations (SD) of symptom burden scores

Of the 558 participants who filled out the symptom section of the survey, all participants endorsed at least one COVID-19 symptoms as designated by the WHO. The number of endorsed symptoms ranged from 1 to 25, with a mean of 10.70 reported symptoms (SD = 5.62). The most frequently reported symptoms were fatigue, cognitive deficits/brain fog, and memory loss. The least frequent symptoms were menstruation problems, new allergies, and abdominal pain. Symptom burden means ranged from 4.73 (SD = 2.88) for menstruation problems to 8.37 (SD = 1.77) for post-exertional malaise (PEM). The total symptom burden sum per individual ranged between 3 and 207 and was on average 72.07 (SD = 43.22). The average symptom burden per individual ranged between 1.15 and 10 and was on average 6.68 (SD = 1.42) Frequencies (%) of symptoms and mean burden scores (M, SD) distributed across symptoms are illustrated in Fig. 2. The symptoms with the highest reported burdens were PEM, fatigue and sleeping problems. The symptoms with the lowest reported symptom burdens were menstruation problems, affected smell/taste, and abdominal pain.

Other symptoms were reported by 21.1% (*n* = 118) of the participants, with the most common being other conditions, eye- or vision-related problems, and breathing-/asthma related symptoms other than shortness of breath. The distribution of other symptoms than those included in the WHO definition of PCC are provided in an additional document file [see Additional file 2].

### Received treatments

**Fig. 3:**
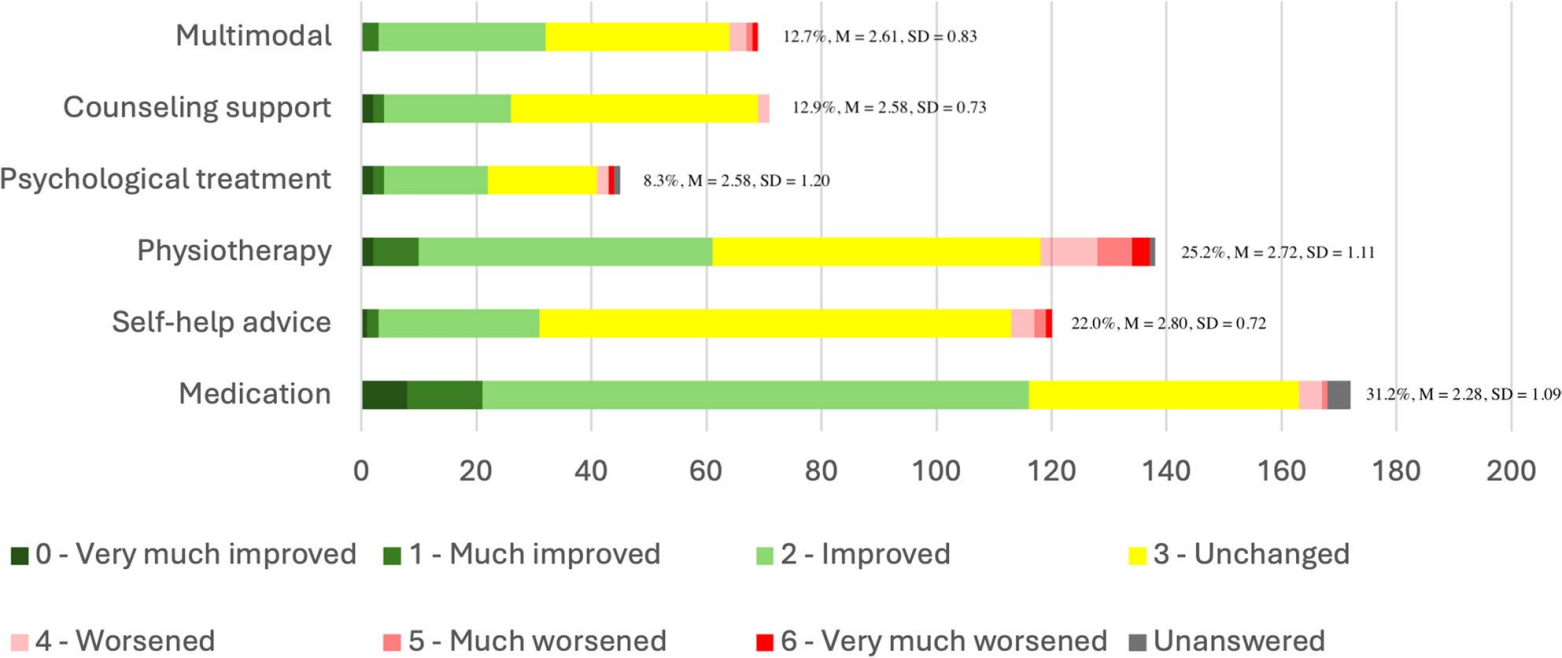
Frequencies of received treatments and distributions of PGIC scores

For the 551 participants that completed the questions about treatments, number of health care visits ranged between 0 and 72 visits during the last 6 months with a mean number of 3.85 health care visits (SD = 7.21). Treatment frequencies and PGIC scores are illustrated in Fig. 3.

Other treatments not included in the prespecified options were reported by 14.5% (*n* = 80). Of these, 26.3% (*n* = 21) had met with an occupational therapist, and 16.3% (*n* = 13) received symptom-targeted treatment. Other treatments also included naprapathy, speech therapy, or lifestyle interventions. 

The group mean PGIC scores for treatments ranged from 2.28 to 2.80 which is between options “3 – unchanged” and “2 – improved”. Although not included in the preselected response options, participants (*n* = 21) who met with an occupational therapist reported an average PGIC value of 1.86 (SD = 0.79) which is between options “2 – improved” and “1 – much improved”. 

### Psychological distress

For the 544 participants who completed the GAD-7, the mean score was 6.38 (SD = 5.37) indicating mild levels of anxiety, and 24.1% (*n* = 131) reporting a score over the 10-point threshold. The PHQ-9 was filled out by 540 participants, with a mean score of 10.53 (SD = 5.93) indicating moderate levels of depression, and 53.5% (*n* = 289) reporting a score over the 10-point threshold. The 2-item ISI was filled out by 539 participants with a mean total score of 4.86 (SD = 2.10), and 42.1% (*n* = 227) reporting a score above the 6-point threshold. At the item level, the mean score was 2.61 (SD = 1.02) for the sleep quality item, indicating being between neutral and dissatisfied with one’s current sleep pattern, and 2.24 (SD = 1.23) for the interference item, indicating functioning being between somewhat worsened and much worsened by sleeping problems.

### Satisfaction with life and functioning

**Fig. 4:**
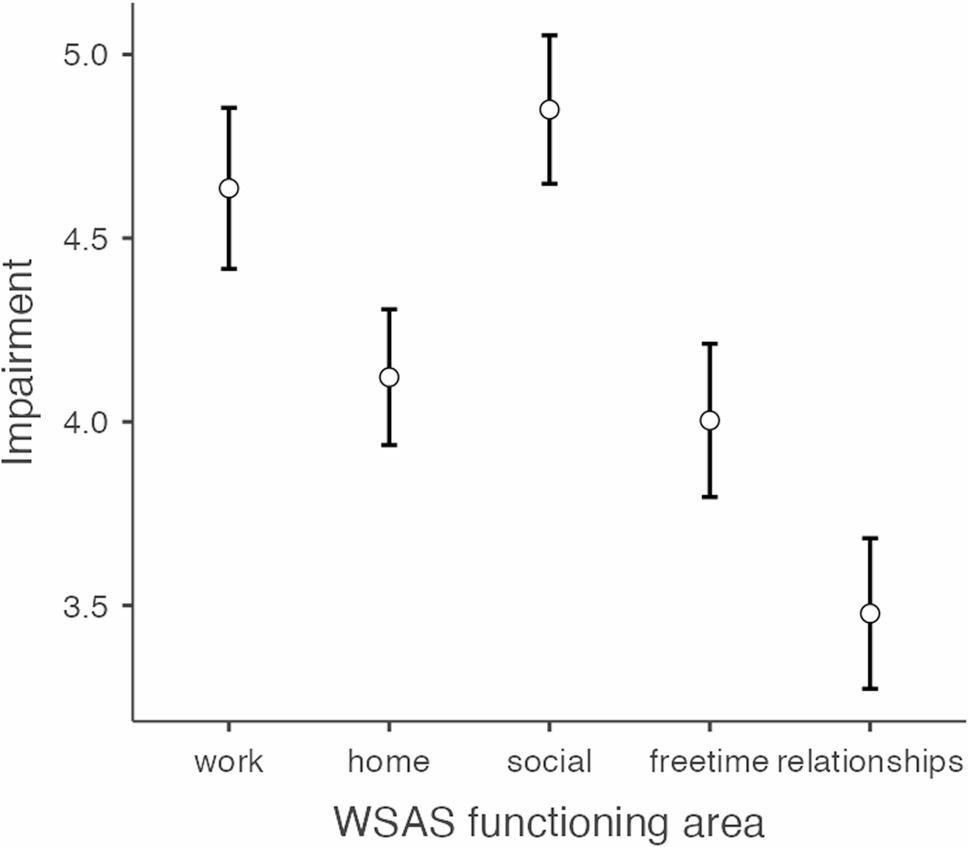
Estimated Marginal Means with 95% confidence intervals (CI) for post-hoc comparisons of impairment between WSAS functioning areas

The SWLS was completed by 529 participants with a mean score of 18.21 (SD = 7.85) indicating being “somewhat dissatisfied” with life. Scores below 15 points, indicating being dissatisfied or extremely dissatisfied with life, were reported by 37.8% (*n* = 200). Respectively, 23.6% (*n* = 125) were dissatisfied with life and 14.2% (*n* = 75) were extremely dissatisfied with life. The WSAS was completed by 527 participants with a mean score of 21.09 (SD = 10.27) indicating severe levels of impairment. WSAS scores above the 20-point threshold for severe impairment were reported by 54.5% (*n* = 287). At the single item level, WSAS mean scores ranged from 3.48 to 4.85. Highest impairment scores were found for social activities (M = 4.85, SD = 2.36) and work/studies (M = 4.64, SD = 2.56). ANOVA showed a within-subject difference for WSAS items, *F*(4, 2104) = 84.2, *p* < .001. Bonferroni-corrected post-hoc comparisons between WSAS items are presented in Fig. 4, showing significant differences in functioning between all items except for the work versus social activities item, and home versus free time item. The largest mean difference identified was between social activities and relationships.

### Correlation analysis

Correlations between demographic variables, COVID-19 history/severity, PCC symptoms/symptom burden, psychological distress, SWL and functioning are fully presented in Table 2. Older participants reported being on sick leave less, and lower functional impairment. Highly educated as well as participants with some form of occupation, and better financial status, reported lower symptom burden and less psychological distress. Additionally, those with higher occupational and financial status reported higher SWL and less functional impairment. Those who had COVID-19 more often reported more PCC symptoms. Being on sick leave correlated with health care visits as well as symptom burden, depression, insomnia, SWL (negatively), and functional impairment. Having sought help for PCC correlated with symptom burden, depression, insomnia, SWL (negatively) and functional impairment, indicating that people with higher impacts from PCC in Sweden are more likely to seek treatment for their problems. Being bedridden and hospitalized due to COVID-19, correlated with number of PCC symptoms, depression and sleeping problems. Symptom burden correlated with psychological distress and depression in particular, SWL (negatively) and functional impairment. Psychological distress, SWL, and functional impairment all correlated with each other.

**Table 2:** Correlations between demographics, COVID-19, PCC symptoms, symptom burden, health care consumption, psychological distress, SWL, functioning

Variable	1.	2.	3.	4.	5.	6.	7.	8.	9.	10.	11.	12.	13.	14.	15.	16.	17.	18.	19.	20.	21.
**1.** Gender^a^	—																				
**2.** Age	0.06	—																			
**3.** Education level^b^	0.11*	− 0.05	—																		
**4.** City size^c^	− 0.06	− 0.13*	0.09	—																	
**5.** Occupation status^d^	− 0.01	− 0.31**	0.07	− 0.03	—																
**6.** Financial status^e^	0.04	0.07	0.15**	− 0.03	0.24**	—															
**7.** Marital status^f^	− 0.02	− 0.05	− 0.02	− 0.05	0.08	0.23**	—														
**8.** Children at home	0.02	− 0.36**	0.20**	0.02	0.14*	− 0.09	0.15**	—													
**9.** Covid episodes^g^	− 0.02	− 0.09	0.04	0.06	0.09	− 0.04	0.04	0.06	—												
**10.** Sick leave extent^h^	0.07	− 0.22**	0.06	0.12*	− 0.12*	− 0.15**	0.03	0.06	0.06	—											
**11.** Sick leave duration^i^	0.17	0.11	0.07	− 0.02	− 0.09	0.05	0.07	− 0.06	− 0.12	—	—										
**12.** Health care visits^j^	0.07	− 0.07	− 0.01	0.09	− 0.06	− 0.09	0.07	0.02	0.01	0.42**	− 0.02	—									
**13.** Bedridden^k^	0.10	0.07	0.01	0.01	− 0.05	− 0.09	− 0.05	0.00	0.05	0.02	0.07	0.03	—								
**14.** Hospitalized^l^	− 0.09	0.14*	− 0.05	0.00	0.01	− 0.01	0.10	− 0.05	− 0.08	− 0.01	− 0.05	0.07	0.13*	—							
**15.** Number of symptoms	0.06	− 0.09	− 0.07	0.07	− 0.13*	− 0.21**	0.07	0.01	0.14**	0.34**	− 0.02	0.29**	0.12*	0.12*	—						
**16.** Symptom burden	0.05	− 0.08	− 0.13*	0.06	− 0.17**	− 0.21**	0.05	0.01	0.09	0.33**	− 0.07	0.29**	0.11	0.12*	0.93**	—					
**17.** GAD-7	− 0.01	− 0.09	− 0.15**	0.05	− 0.11*	− 0.20**	− 0.03	− 0.01	0.01	0.10	− 0.22*	0.10	0.08	0.14*	0.35**	0.41**	—				
**18.** PHQ-9	− 0.03	− 0.04	− 0.20**	0.05	− 0.24**	− 0.28**	− 0.09	− 0.06	0.00	0.24**	− 0.06	0.14**	0.14*	0.12*	0.45**	0.53**	0.68**	—			
**19.** ISI-2	− 0.05	0.05	− 0.15**	0.07	− 0.17**	− 0.19**	− 0.08	− 0.03	0.03	0.14*	− 0.13	0.14*	0.16**	0.15**	0.31**	0.37**	0.33**	0.54**	—		
**20.** SWLS	0.04	0.03	0.05	− 0.06	0.26**	0.34**	0.14**	0.04	0.03	− 0.23**	0.07	− 0.18**	− 0.12*	-0.07	− 0.31**	− 0.34**	− 0.39**	− 0.49**	0.37**	—	
**21.** WSAS	0.01	− 0.17**	− 0.03	0.09	− 0.27**	− 0.27**	− 0.03	0.08	0.00	0.50**	0.02	0.33**	0.13*	0.11	0.51**	0.57**	0.36**	0.63**	0.43**	− 0.51**	—

*GAD-7* Generalized Anxiety Disorder 7-item scale, *PHQ-9* Patient Health Questionnaire, *ISI-2* Insomnia Severity Index 2-item Index 2-item version, *SWLS *Satisfaction With Life Scale, *WSAS *Work and Social Adjustment Scale

Significant correlations are **bold**

*p < .01; **p < .001

a Gender; 1 = woman, 0 = other genders than woman (man, non-binary, unsure, prefer not to answer)

b Education level; 0 = lower (elementary, high school, vocational- or folk high school, university level courses), 1 = higher (bachelor’s degree, master’s degree, and PhD)

c City size; 1 = medium to large cities (large cities, commuting municipalities near large cities, medium-sized towns), 0 = small city/town and rural (commuting municipalities near medium-sized towns, commuting municipalities with a low commuting rate near medium-sized towns, small towns, commuting municipalities near small towns, rural municipalities, rural municipalities with a visitor industry)

d Occupational status; 1 = Occupation (studying, employed, self-employed, and Job training or similar via e.g. Swedish Public Employment Service), 0 = No occupation (unemployed, sick leave for a longer period of time (> 60 days), parental leave, disability pension, and retired)

e Financial status; 1 = Higher (good, very good), 0 = Lower (sufficient, bad, very bad)

f Marital status; 1 = partnered (married, living with partner, and partnered living apart) and 0 = single (single, divorced, and widowed)

g Values 0 and 10 were treated as outliers and removed in the analysis

h Sick leave extent; 0 = Not on sick leave, 1 = On sick leave (25%-, 50%-, 75%-, and 100% sick leave)

i Sick leave duration; 0 = short-term sick leave (< 2 weeks, ≥ 2 weeks, ≥ 1 month), 1 = long-term sick leave (≥ 2 months, ≥ 3 months, ≥ 6 months, ≥ 12 months)

j Health care visits; 0 = no health care visits, 1 = at least one health care visit

k Bedridden; 0 = no, 1 = yes

l Hospitalized; 0 = no hospital care, 1 = hospital care (‘Yes, not in the ICU’, ‘Yes, in the ICU’)

None of the clinical variables, including PCC symptoms or burden, psychological distress, SWL, nor functioning, differed based on gender. Age was not associated with symptom burden, nor number of symptoms. Number of COVID-19 infections was not associated with sick leave, health care visits, being bedridden, psychological distress, SWL, or functional impairment. Being on sick leave for PCC was not associated with COVID-19 severity, and sick leave duration was not related to health care consumption, COVID-19 severity, symptom burden, depression, sleep, SWL, or functional impairment. 

## Discussion

While aspects of PCC etiology, conceptualization, and treatment are inconclusive, debated, and uncertain, the experience of symptoms and impact on individual people appear substantial. The main findings in this study include high average numbers of reported symptoms, as well as typically high symptom burden scores, in people experiencing PCC. Symptoms of fatigue and cognitive impairments were the most frequently reported symptoms, and the symptoms with the highest average burden scores were post-exertional malaise (PEM), fatigue, and cognitive impairments. Medication, self-help advice, and physiotherapy were reported more frequent than other treatments for PCC. More than half of those receiving medication treatment reported it improved their problems, although a few respondents reported that these made things worse. Those who received physiotherapy reported worsening problems to a greater extent than other treatments, while at the same time a relatively large proportion of participants reported improvement. There was mild anxiety reported, with a mean below the clinical cutoff, and somewhat greater depression, with a mean above the cutoff. There were, on average, subclinical but notable sleeping problems, a “somewhat” level of dissatisfaction with life, and substantial levels of impairment, especially in work and social activity. Correlation analyses revealed several relationships worth noting, and exploring in more detail in future studies.

Several findings from this study align with previous PCC research but also reflect the complexity and heterogeneity of this research area. For instance, gender has been identified as a risk factor for developing neurological symptoms of PCC though the primary studies report varying distributions of women/female and men/male participants [34]. However, gender was not evenly distributed in this study, with a much higher proportion of women, meaning associations with gender should be interpreted with some caution. The predominance of fatigue and cognitive impairments is consistent with prior research on PCC (e.g. [35, 36, 37]). and similar to symptoms occurring in conditions such as Myalgic Encephalomyelitis/Chronic Fatigue Syndrome (ME/CFS) and stress-related conditions such as exhaustion disorder.

The finding that younger age predicted greater functional impairment contrasts with previous findings that identified older age as a risk factor for such impairment [38, 34]. This may however reflect the sampling strategy and study design which could obscure some wider pattern. Findings indicating that higher SES predicts lower impact of PCC, reduced psychological distress, less functional impairment, and higher SWL are partially consistent with previous Swedish studies [38]. However, direct comparisons are limited due to differences in measures used. Participants report being somewhat dissatisfied with life is comparable to baseline scores in a clinical trial with a PCC sample [39], and reporting severe functional impairment is in line with Walker et al. [40]. Finally, the relationships among the clinical variables are in line with previous findings of psychological distress being highly comorbid with the wider set of symptoms (e.g. [41]).

While many participants reported psychological distress, they reported mild levels of anxiety on the group-level. A notably high share of participants also reported no anxiety, i.e. scored 0 on the GAD-7. The reason for this is unknown, but could be explored via attempts to replicate and conduct qualitative interviews in future research. The association between young age and functional impairment should be furtherly explored but results from this sample might be slightly indicative of higher needs of support in this part of the population. A possible explanation for this difference may be less developed support networks around young people, and fewer experiences of navigating setbacks in life. Entering university or occupation may also be life transitions that make people more vulnerable to effects of changes in health of this kind. Further, while the relationships between SES and clinical outcomes need further exploration, and many of them are small, they might shed light on possible inequalities in socioeconomic resources in the context of PCC, and that the condition might be more manageable for people in better circumstances. Worth keeping in mind is that most participants in this study were highly educated, had an occupation, and a large proportion perceived their financial status as good or very good. We are unable to determine whether sample characteristics reflect true demographics for people with PCC or whether respondents represent a selected subset of this larger group.

Results regarding received treatments in this study provide information on what *modalities* were most common and perceived as most helpful. Of course, results like these should not be taken as indication of treatment effectiveness or reflecting assumptions of cause and effect. The relatively higher number of participants reporting worsening from physiotherapy compared to medication treatment may be explained by different expectations for outcomes or side-effects from treatment, but this is speculative [42].

Both previous findings, and results in this study, document complex symptom experiences and substantial impact of symptoms on daily functioning. Such findings suggest that SWL and functional impairment are highly relevant. These variables may be desirable outcomes of change for future treatments. In this study, an ANOVA revealed a within-subject difference for WSAS items with the biggest difference being between relationships and social activities which may appear surprising because relationships typically include social activities, and vice versa. However, the relationships item in the WSAS refers to creating and maintaining relationships and uses examples from closer ones like family, partner and friends. The social activities item on the other hand, uses examples of participation in social activities with people of varying closeness (e.g. “meeting new people”, “going to parties”). These results are interesting and suggest that a finer-grained analyses of social impact could be fruitful.

This study identifies several findings worth exploring further, as well as methodological considerations for future studies. Given that PCC has been shown to be a heterogeneous, and in some cases complex and highly burdensome condition, future research should prioritize identifying variables contributing to the development and maintenance of the condition. Identifying clusters of symptoms, or other relevant modifiable variables feeding into the condition, may be another way of approaching PCC clinically. For psychological treatments supporting wellbeing and functioning for people with PCC, these variables should be included as targets in clinical trials. In the light of findings about received treatments, future research should explore negative effects in PCC treatments. Finally, treatment evaluations at the intervention or process of change level are warranted to better understand “what treatment, by whom, is most effective for this individual with that specific problem, under which set of circumstances, and how does it come about?” [45].

This study has several strengths. First, a large number of participants were recruited. One might surmise from the sample size that the prevalence of PCC in Sweden appears to be substantial. A large proportion of the participants that started the survey also completed it, and none of the survey sections stood out in terms of attrition. It is also worth mentioning the high interest for the social media advertising of the study: 1.75% of the Swedish population was reached by the ads (*n* = 185,491). For these, the *click through rate* (CTR), or the ratio between clicks on the ad and people reached, was 2.80% (*n* = 13,029). For context, average CTR scores for health (and fitness) are around 0.59% (e.g. [46]). In addition, the survey covered many aspects of PCC and its impact across different domains assessed with well-validated measures. This makes results comparable to previous research and mirrors the authors’ own recommendations on addressing PCC from multiple perspectives such as biomedical, psychological, and social. A third strength was the liberal inclusion criteria, meaning that results are likely to be more ecologically valid and generalizable to a broader population, compared to results if we had required a formal diagnosis of PCC. We believe this study provides an informative snapshot of PCC impact in a large number of people, in multiple domains of health and wellbeing, in Sweden.

This study includes several important limitations. First, while our sample size is relatively large and people were recruited from a wide range of locations in Sweden, we cannot claim that our recruitment produced a representative sample and it does not allow us to precisely determine the prevalence of PCC in Sweden. Another limitation of this study is that it is an observational study and does not allow us to infer cause and effect. The choice to dichotomize variables to handle non-normal distributions and facilitate uniform analyses may have been on the expense of a loss in details contained in the original information. Using conditional follow-up questions may have created some ambiguity or missing data points. Trade-offs like these are not unique to this survey study, though important to discuss, especially in the context of a relatively new research topic like PCC.

We note that the measure yielding the composite symptom burden variable was newly devised by us. The symptom burden score was created by summing all burden scores for every symptom. Since these were follow-up questions dependent on whether symptoms were endorsed, much of the variance in the composite variable might be explained by number of symptoms. This might in turn make the content validity of the burden variable questionable. Another concerning aspect of the total symptom burden sum per individual measure is the many possible combinations of scores. High scores on the measure can be reflective of either a high number of endorsed symptoms with low burden scores, few endorsed but burdensome symptoms, or other combinations. A post-hoc sensitivity analysis showed that when the total symptom burden sum was divided by number of symptoms, creating an average burden score per individual ranging from 0 to 10, the relationships between symptom burden and several other variables changed. A comparison of the relationships between the two reflections of symptom burden and other variables is provided in an additional document file [see Additional file 3]. What is clear is that there is variance in the data which is not explained by number of symptoms. At the same time, it is not obvious that number of symptoms should be disregarded when assessing symptom burden in PCC. For instance, one might argue that a person with many symptoms with low burden scores is differently affected by PCC than someone with few symptoms but with higher burden scores although their total burden score sums are similar. The two variables seem to measure different aspects of symptom burden in PCC.

Another limitation in this study is potential recall bias, since many questions considered events that might have occurred several months or even years ago, such as being bedridden due to COVID-19. Regarding the bedridden variable, there are other potential limitations reflected in the wide range of reported days with some participants reporting having been bedridden due to COVID-19 for five years. While this is possible, we encourage cautious interpretation of these results. The standardized questionnaires may also have been subject to this bias since they rely on self-report from relatively extended periods of time, such as “during the past two weeks”, possibly making data less reliable. This issue is clearly not unique to this study, as the use of questionnaires with wider time frames is a typical measurement method in clinical research. Finally, using a single time point for measurement per participant prevented examination of possible intraindividual temporal variations of the condition. Recruitment took place during five months and six days. In a Swedish context, seasonal variation is substantial and likely represents a source of variability in health outcomes. The research area of seasonality in similar fields such as chronic pain is however ambiguous (see Abeler et al. [47] for an overview).

We have several recommendations for future research. While it is tempting to focus on an underlying cause of symptoms and then plan to match specific treatments with this cause or causes [43], another approach is possible. Previous research shows beneficial effects of cognitive training on stress-related cognitive deficits, for example [44], and while such approaches may work for stress-related conditions, the same need not necessarily be true for conditions like PCC. Given the complexity and multi-symptom nature of PCC, one could adopt a process-based approach and apply treatment that is able to simultaneously address multiple underlying causes or mechanisms, and multiple symptoms or impacts. One such approach is Acceptance and Commitment Therapy (ACT), which is grounded in the psychological flexibility (PF) model [48]. We know that ACT is an effective treatment for increasing quality of life and functioning in the context of chronic pain [49], cancer [50] and other chronic health conditions [51]. It also appears feasible for patients with chronic fatigue (ME/CFS), a condition quite similar to PCC, as a previous study found that disability was reduced (d = 0.80) and that ACT was both safe and accepted by participants [52].

Furthermore, methodological improvements are warranted in future research as well. First, it seems important to reduce the effect of recall bias in the data. One way of doing this is to use ecological momentary assessment (EMA). In contrast to traditional full-length measures, typically asking participants/patients to report on outcomes based on longer periods of time (weeks, months), EMA means using fewer items, more frequently, and the time frame for questions is typically narrower. Not only does this increase the reliability of responses, but it also provides researchers with a higher temporal resolution for processes of change, sometimes not captured in conventional monthly, or weekly measurements. It is a well-established notion that results from individual cases are not necessarily generalizable to the group-level while the reverse is much less appreciated; results from studies evaluated at the group-level do not necessarily generalize to individuals, or what is called a failure in the assumption of *ergodicity* [53]. Another methodological consideration for future studies and treatment evaluations is the choice of measurements. As PCC is shown to have multilevel and multidomain impact, affecting many different areas of people’s lives, future studies should include outcome measures that include domains such as health, healthcare use, participation in life, social role performance, and other aspects of well-being, to suggest a few.

## Conclusion

Five years after the COVID-19 pandemic, post-covid condition (PCC) appears to remain as a health problem globally and, as is observed here, in Sweden. Despite methodological limitations inherent in observational survey studies, this study shows a heterogeneous and high impact of PCC in Sweden for many people, in several domains of health and wellbeing, that should be furtherly explored and addressed. Future research should prioritize identifying modifiable predictors and potential influences on PCC and related impacts, particularly those that can be incorporated in treatment development. A suitable one may be psychological flexibility (PF).

## Supplementary Information

Additional file 1: English translation of survey. An English translation of the survey items developed for this study.

Additional file 2: Supplementary table 1. Other symptoms reported than those provided in the set list of symptoms.

Additional file 3: Supplementary table 2. Correlations with other variables for total PCC burden sum score and average PCC burden per individual, respectively.

## Data Availability

Datasets used in this study are available from the corresponding author upon reasonable request.

## Abbreviations

ACT: Acceptance and Commitment Therapy
CBT: Cognitive Behavioral Therapy
CTR: Click through rate
EMA: Ecological momentary assessment
GAD-7: Generalized Anxiety Disorder-7
ISI-2: Insomnia Severity Index
ME/CFS: Myalgic Encephalomyelitis/Chronic Fatigue Syndrome
PCC: Post-covid condition
PEM: Post-exertional malaise
PF: Psychological flexibility
PGIC: Patient Global Impression of Change
PHQ-9: Patient Health Questionnaire
RedCAP: Research Electronic Data Capture
SES: Socioeconomic status
SWL: Satisfaction with life
SWLS: Satisfaction with Life Scale
WHO: World Health Organization
WSAS: Work and Social Adjustment Scale
