# Supplementary material for: Impacts of Post-Covid Condition (PCC) in Sweden: a cross-sectional observational survey study
Source: BMC Public Health. 2026 May 12;26:1525. doi: 10.1186/s12889-026-27720-7 (PMC13162529; doi:10.1186/s12889-026-27720-7)
Supplement: Supplementary file 1 — Additional file 1: English translation of survey. An English translation of the survey items developed for this study. [file 12889_2026_27720_MOESM1_ESM.docx]

**English translation of survey**

**What gender do you identify as?**

- Woman
- Man
- Non-binary
- Unsure
- Prefer not to answer
- Other alternative: [free text]

**How old are you?**

[free text]

**What is your highest completed form of education?**

- Elementary school
- High school
- Vocational education
- Individual courses at university level
- Three-year university education
- Five-year university education
- PhD
- Other: [free text]

**Please enter your marital status**

- Single
- Partnered, living together
- Partnered, living apart
- Married
- Divorced
- Widow/-er

**How many children do you have?**

[free text]

**Do you have children under 18 still living at home with you?**

- Yes
- No

**Where do you live? Please write the name of the municipality.**

[list with all Swedish municipalities]

**How is the financial situation of your household?**

- Very good
- Good
- Sufficient
- Poor
- Very poor

**What is your main occupation?**

- Unemployed
- Student
- Employed
- Self-employed
- On parental leave
- Disability pension
- On sick leave for a longer period of time (>60 days)
- Internship or similar via Swedish Public Employment Service
- Retired
- Other

**Are you on sick leave for post covid to any extent?**

- No
- Yes, 25 %
- Yes, 50 %
- Yes, 75 %
- Yes, 100 %

**For how long have you been on sick leave for post covid?**

- Less than 2 weeks
- 2 weeks or longer
- 1 month or longer
- 2 months or longer
- 3 months or longer
- 6 months or longer
- 12 months or longer

**The following questions are about the covid-19 infection.**

**Have you been infected with covid-19?**

- Yes
- No
- Unsure

**How do you know it was covid-19 you had?**

- I was tested by health care professionals (e.g at a primary care unit, at a hospital, in a testing tent, at retirement home)
- I took a self-test
- I wasn’t tested, but the symptoms I had indicated that it was covid-19
- Other

**Please specify if you picked the alternative “Other”:** [free text]

**How many times have you had covid-19?**

[free text]

**Have you ever been bedridden due to a covid-19 infection?**

- No
- Yes

**Please estimate the number of days you’ve been bedridden due to a covid-19 infection, in total.**

[free text]

**Have you received hospital healthcare for covid-19?**

- No
- Yes, but not at an intensive care unit (ICU)
- Yes, at an ICU

**The following questions are about new and/or persisting health problems after covid-19.**

**Post covid condition is associated with many different symptoms. Please mark which of the following symptoms you’ve had during at least two months, and which arose or persisted at least three months after the covid-19 infection.**

- Abdominal pain
- Menstrual and period problems
- Altered smell/taste
- Anxiety
- Blurred vision
- Chest pain
- Cognitive dysfunction/brain fog
- Cough
- Depression
- Dizziness
- Fatigue
- Intermittent fever
- Gastrointestinal issues (diarrhea, constipation, acid reflux)
- Headache
- Memory issues
- Joint pain
- Muscle pain/spasms
- Neuralgias
- New onset allergies
- Pins and needles sensations
- Post-exertional malaise
- Shortness of breath
- Sleep disorders
- Tachycardia/palpitations
- Tinnitus and other hearing issues
- Other
- None of the above

**Please specify if you checked the alternative “Other”:** [free text]

**To what extent are you affected by [checked symptom 1, 2, 3, … , n]?**

[slider from *0 – Not at all* to *10 – Extremely*]

**During the last 6 months, how many health care visits have you made related to post covid?**

[free text]

**What type of treatment have you received for post covid?**

- I haven’t received any treatment
- Medical treatment
- Self-care advice from physician and/or nurse
- Physiotherapy
- Structured psychological treatment from lic. Psychologist and/or lic. psychotherapist
- Psychosocial support from e.g. counselor or behavioral scientist
- Multimodal treatment/rehabilitation (i.e. at least two different professions involved in the same treatment)
- Other, please specify: [free text]

**How have your problems changed after [checked treatment 1, 2, 3, …, n],?**

- 0 Very much improved
- 1 Much improved
- 2 Minimally improved
- 3 No change
- 4 Minimally worsened
- 5 Much worsened
- 6 Very much worsened

**[GAD-7, 7 items]**

**[PHQ-9, 9+1 items]**

**[ISI-2, 2 items]**

**[SWLS, 5 items]**

**[WSAS, 5 items]**

**[Psychological flexibility (PF), 6+95 items]**

**[Qualitative items]**

**We might conduct in-depth studies in the future about the experience of living with post covid. Would it be okay for you to be contacted by us and asked to participate in such a study? If so, please enter your contact information in the following question.**

- Yes
- No

**Please enter your contact information to declare your interest in participating in future studies of post covid. Your contact information is stored according to GDPR, no longer than needed for the study’s purpose.**

[free text]
