# Supplementary material for: Impacts of Post-Covid Condition (PCC) in Sweden: a cross-sectional observational survey study
Source: BMC Public Health. 2026 May 12;26:1525. doi: 10.1186/s12889-026-27720-7 (PMC13162529; doi:10.1186/s12889-026-27720-7)
Supplement: Supplementary file 2 — Additional file 2: Supplementary table 1. Other symptoms reported than those provided in the set list of symptoms. [file 12889_2026_27720_MOESM2_ESM.docx]

| **Supplementary table 1**  *Other symptoms reported than those provided in the set list of symptoms* | | | | |
| --- | --- | --- | --- | --- |
| **Symptom** | **N** | | | **%** |
| Other conditions |  | 16 |  | 2.9 |
| Eye- or vision-related symptoms |  | 15 |  | 2.7 |
| Breathing/Asthma |  | 14 |  | 2.5 |
| Skin problems |  | 13 |  | 2.3 |
| Temperature regulation difficulties |  | 11 |  | 2.0 |
| Throat problems |  | 9 |  | 1.6 |
| Affected blood pressure/pulse/oxygen in blood |  | 8 |  | 1.4 |
| POTS |  | 8 |  | 1.4 |
| More susceptible/sensitive to infections |  | 6 |  | 1.1 |
| Mucus |  | 5 |  | 0.9 |
| Nausea/vomiting |  | 5 |  | 0.9 |
| Swelling |  | 5 |  | 0.9 |
| Appetite/weight change |  | 4 |  | 0.7 |
| Hair loss |  | 4 |  | 0.7 |
| Sensory sensitivity |  | 4 |  | 0.7 |
| Pressure over chest/back |  | 3 |  | 0.5 |
| Affected dreaming/nightmares |  | 2 |  | 0.4 |
| Feet problems |  | 2 |  | 0.4 |
| Lymph nodes |  | 2 |  | 0.4 |
| Sinus-related |  | 2 |  | 0.4 |
| Tremors/cramps |  | 2 |  | 0.4 |
| Weakness |  | 2 |  | 0.4 |
| Chills |  | 1 |  | 0.2 |
| Depersonalization/derealization |  | 1 |  | 0.2 |
| Emotionally numb |  | 1 |  | 0.2 |
| Fainting |  | 1 |  | 0.2 |
| Malaise |  | 1 |  | 0.2 |
| Speaking difficulties |  | 1 |  | 0.2 |

*Note.* Symptoms are sorted into categories based on what is affected to provide a better overview.
