# Supplementary material for: Impacts of Post-Covid Condition (PCC) in Sweden: a cross-sectional observational survey study
Source: BMC Public Health. 2026 May 12;26:1525. doi: 10.1186/s12889-026-27720-7 (PMC13162529; doi:10.1186/s12889-026-27720-7)
Supplement: Supplementary file 3 — Additional file 3: Supplementary table 2. Correlations with other variables for total PCC burden sum score and average PCC burden per individual, respectively. [file 12889_2026_27720_MOESM3_ESM.docx]

**Supplementary table 2**

*Correlations with other variables for total PCC burden sum score and average PCC burden per individual, respectively.*

| **Variable** | **Total burden sum score (0-250)** | **Average burden per individual (0-10)** |
| --- | --- | --- |
| **1.** Gender^a^ | .05 | .06 |
| **2.** Age | -.08 | .03 |
| **3.** Education level^b^ | **-.13*** | **-.18**** |
| **4.** City size^c^ | .06 | .00 |
| **5.** Occupation status^d^ | **-.17**** | **-.15**** |
| **6.** Financial status^e^ | **-.21**** | -.09 |
| **7.** Marital status^f^ | .05 | -.03 |
| **8.** Children at home | .01 | .01 |
| **9.** Covid episodes^g^ | .09 | -.08 |
| **10.** Sick leave extent^h^ | **.33**** | .09 |
| **11.** Sick leave duration^i^ | -.07 | -.15 |
| **12.** Health care visits^j^ | **.29**** | .07 |
| **13.** Bedridden^k^ | .11 | .02 |
| **14.** Hospitalized^l^ | **.12*** | .06 |
| **15.** Number of symptoms | **.93**** | .07 |
| **16.** Total burden sum score (0-250) | - | **.39**** |
| **17.** GAD-7 | **.41**** | **.22**** |
| **18.** PHQ-9 | **.53**** | **.30**** |
| **19.** ISI-2 | **.37**** | **.23**** |
| **20.** SWLS | **-.34**** | **-.15**** |
| **21.** WSAS | **.57**** | **29**** |

*Note.* GAD-7=Generalized Anxiety Disorder 7-item scale; PHQ-9=Patient Health Questionnaire; ISI-2=Insomnia Severity Index 2-item Index 2-item version; SWLS=Satisfaction With Life Scale; WSAS = Work and Social Adjustment Scale.

*p < .01; **p < .001.

a Gender; 1=woman, 0=other genders than woman (man, non-binary, unsure, prefer not to answer).

b Education level; 0=lower (elementary, high school, vocational- or folk high school, university level courses), 1=higher (bachelor’s degree, master’s degree, and PhD).

c City size; 1=medium to large cities (large cities, commuting municipalities near large cities, medium-sized towns), 0=small city/town and rural (commuting municipalities near medium-sized towns, commuting municipalities with a low commuting rate near medium-sized towns, small towns, commuting municipalities near small towns, rural municipalities, rural municipalities with a visitor industry).

d Occupational status; 1=Occupation (studying, employed, self-employed, and Job training or similar via e.g. Swedish Public Employment Service), 0=No occupation (unemployed, sick leave for a longer period of time (>60 days), parental leave, disability pension, and retired)

e Financial status; 1=Higher (good, very good), 0=Lower (sufficient, bad, very bad)

f Marital status; 1=partnered (married, living with partner, and partnered living apart) and 0=single (single, divorced, and widowed).

g Values 0 and 10 were treated as outliers and removed in the analysis.

h Sick leave extent; 0=Not on sick leave, 1=On sick leave (25%-, 50%-, 75%-, and 100% sick leave).

i Sick leave duration; 0=short-term sick leave (< 2 weeks, ≥ 2 weeks, ≥1 month), 1=long-term sick leave (≥ 2 months, ≥ 3 months, ≥ 6 months, ≥ 12 months).

j Health care visits; 0=no health care visits, 1=at least one health care visit.

k Bedridden; 0=no, 1=yes.

l Hospitalized; 0=no hospital care, 1=hospital care (‘Yes, not in the ICU’, ‘Yes, in the ICU’).
